# Capturing fingerprints of conical intersection: Complementary information of non-adiabatic dynamics from linear x-ray probes

**DOI:** 10.1063/4.0000093

**Published:** 2021-05-03

**Authors:** Deependra Jadoun, Mahesh Gudem, Markus Kowalewski

**Affiliations:** Department of Physics, Stockholm University, Albanova University Centre, SE-106 91 Stockholm, Sweden

## Abstract

Many recent experimental ultrafast spectroscopy studies have hinted at non-adiabatic dynamics indicating the existence of conical intersections, but their direct observation remains a challenge. The rapid change of the energy gap between the electronic states complicated their observation by requiring bandwidths of several electron volts. In this manuscript, we propose to use the combined information of different x-ray pump-probe techniques to identify the conical intersection. We theoretically study the conical intersection in pyrrole using transient x-ray absorption, time-resolved x-ray spontaneous emission, and linear off-resonant Raman spectroscopy to gather evidence of the curve crossing.

## I. INTRODUCTION

Conical intersections (CIs),^1–3^ though once considered rare, govern the outcome of many photochemical reactions, according to modern photochemistry.^4–6^ They appear in a molecule when two or more potential energy surfaces (PESs) cross and enable the possibility of electronic de-excitation without radiative emission. These non-radiative transitions result in the breakdown of the Born–Oppenheimer approximation. Consequently, the electronic and nuclear degrees of freedom are strongly coupled in the vicinity of the CI. It is now well established that the CIs play a vital role in several photochemical processes such as photosynthesis,^7^ primary event of vision,^8^ photochemical formation of DNA lesions,^9^ and photochemistry of individual nucleobases.^10^ However, the direct experimental detection of CIs in molecular systems is still challenging. This is mainly because the energy gap between PESs near the CI decreases rapidly. Ultrashort pulses with adequate resolution in both energy and time domain are required to observe them.

With the advent of x-ray lasers, it is possible to probe such fast processes that occur on the timescale of femtoseconds and attoseconds using ultrafast pump-probe spectroscopy.^11–21^ It has been recently shown experimentally that attosecond transient absorption spectroscopy (ATAS) can aid the observation of non-adiabatic processes.^22–25^ Time-resolved photo-electron spectroscopy^26,27^ and x-ray diffraction^28^ have also been proposed to study the behavior of a system in the vicinity of a CI.

Spectroscopic techniques that involve attosecond pulses provide a high resolution in the time-domain and cover a broad range of frequencies, making them a promising tool to measure the rapidly varying energy gap that comes with CIs. However, their experimental availability can be a limiting factor.

In the following, we show theoretically how a combination of femtosecond based x-ray probe methods may be used to detect the non-adiabatic dynamics in a molecule. Our chosen example molecule, pyrrole, exhibits a CI in the photodissociation pathway of the hydrogen atom. The branching ratio between the electronic states in the vicinity of the CI is on the order of a few percent, making it a rather challenging example.^29^ The goal of the present work is to show that it is possible to locate a CI even when there is an unbalanced branching of the electronic state population in its vicinity, using widely used femtosecond x-ray probe methods. A purely diabatic or purely adiabatic population transfer poses a challenge for spectroscopic x-ray techniques to identify the existence of the second electronic state involved in the CI. Three different techniques have been employed to study the photo dynamics of pyrrole involving a CI between ground and excited electronic states. The methods used here are as follows: time-resolved x-ray absorption spectroscopy (XAS), x-ray spontaneous emission spectroscopy (XSES), and a recently proposed technique, transient redistribution of ultrafast electronic coherence in attosecond Raman signals (TRUECARS). We briefly introduce the features of the methods used in this manuscript in the following.

In the case when there is an unbalanced branching, one needs to look for the indirect signatures of a CI to detect its occurrence. We construct the transient XAS spectrum using femtosecond x-ray probe-pulses to observe the change in transition dipole-moment in the vicinity of a CI in pyrrole. Femtosecond pulses are sufficiently narrow in the frequency domain (≤1 eV, as compared to attosecond pulses) to selectively probe the transitions, which are distributed over a few electron volts. The intensity of the XAS signal depends on the valence-to-core transition dipole moments that the probe pulse is resonant with, as well as the population of the valence states. It is possible to construct XAS spectra using attosecond pulses, but it may contain signatures originating from other resonant valence-to-core transitions, further complicating the interpretation of the spectrum.

Time-resolved XSES^30,31^ does not solely depend upon the branching of the wave packet and may provide additional information about the curve crossing. In the XSES spectrum, the energy gap in the vicinity of the CI may be observed directly independent of the population of the involved valence states and is expected to yield information about the shape of the PESs involved. In time-resolved XSES, pyrrole is excited to a nitrogen 1s core-hole state, which is followed by the spontaneous emission of a photon. The 1s core-hole states of nitrogen have a lifetime of ≈7 fs,^32–34^ and the spontaneous emission takes place within this time period after excitation.

Probing the creation of electronic coherences in the vicinity of CI provides a more direct signature of CIs. Several x-ray based spectroscopic techniques have been proposed theoretically to observe this phenomena.^27,35–37^ One such scheme is TRUECARS,^38–40^ which is based on an off-resonant linear Raman process. In this scheme, a hybrid probe-pulse sequence is used instead of a single probe pulse to construct the signal where the redistribution of photons maps out the time varying energy gap via the Raman shift. TRUECARS is only sensitive to the coherences and is thus expected to yield accurate information about the passage through the CI. However, to obtain the maximum intensity in the signal, a balanced branching at the CI is required. The result from TRUECARS signal can be considered confirmation for the other two methods. The use of hybrid pulses in TRUECARS and homodyne detection scheme makes it experimentally more challenging, whereas XAS and XSES have been used successfully earlier in experiments to study photochemical processes.^41,42^

We show that the three methods deliver complementary information that can be used to observe the direct (generation of vibronic coherence and state resolved spectra) and indirect (change in the transition dipole moments) effects of a CI between two states in the pyrrole molecule. The paper is structured as follows: Sec. II presents the details of the system used for the signal calculations. In Sec. III, the analytical expressions for spectroscopic signals and Hamiltonian for the various pump–probe methods are discussed. Section IV contains the details about the software and programs used to carry out the simulations. In Sec. V, spectra calculated using different pump–probe methods are presented along with the discussion. Finally, the main conclusions drawn by comparing the results from used methods are presented in Sec. VI.

## II. MODEL

### A. Photochemistry of pyrrole

Pyrrole is a nitrogen-containing five-membered heterocyclic aromatic compound, which has been used extensively as a model system for exploring non-adiabatic dynamics.^29,43–48^ The low-lying bright states of pyrrole emerge around 6 eV.^49,50^ It has been challenging to assign the corresponding peaks in the ultra violet (UV) absorption spectrum. This is mainly due to the high density of energy levels lying around the bright states.^51,52^

The UV-induced photochemistry of pyrrole involves an NH detachment process through the manifold of four lowest singlet excited states, ^1^A_2_($\pi \sigma^{*}$), ^1^B_1_($\pi \sigma^{*}$), ^1^B_2_($\pi \pi^{*}$), and ^1^A_1_($\pi \pi^{*}$).^29,53^ The two $\pi \pi^{*}$ states have been assigned to the above-mentioned absorption bands around 6 eV. The molecules in these bright states undergo non-radiative decay to the $\pi \sigma^{*}$ states along the out-of-plane ring deformation coordinate.^54^ The latter states have been found to be repulsive along the NH stretching mode.^44,55,56^ The ring deformation mechanism was also shown to be feasible on the $\pi \sigma^{*}$ states.^54^ Along the dissociation process, the ground state energy increases and forms a CI between *S*_0_ and $\pi \sigma^{*}$ states.^44^ The nature of the $\sigma^{*}$ orbital was also shown to be varied during the detachment process. At the Franck–Condon (FC) geometry, it is of the 3s Rydberg type, which changes to valence $\sigma^{*}$ upon NH stretching and eventually becomes a 1s orbital of the hydrogen atom. This Rydberg-to-valence orbital transformation rationalizes the presence of the dissociation barrier on the $\pi \sigma^{*}$ states.^44^ The photodissociation dynamics of pyrrole involving *S*_0_/$\pi \sigma^{*}$(^1^A_2_ and ^1^B_1_) CIs have been theoretically investigated by Domcke and co-workers.^29^ The study employs the vertical excitation model and simulates the time-dependent dynamics of pyrrole on each $\pi \sigma^{*}$ state separately. Recently, optical cavities have been found to be hugely influencing the photolysis reaction dynamics in the $\pi \sigma^{*}$(^1^B_1_) state.^57^ The present work considers the photodynamics of pyrrole in the $\pi \sigma^{*}$(^1^B_1_) state, referred to as just $\pi \sigma^{*}$ in the rest of the paper, as a model for the study. We have employed a simplified model by considering only the stretching coordinates corresponding to the NH bond. This reduced dimensionality model has been found to be describing the photofragmentation features of pyrrole qualitatively.^29^

The main objective of the paper is to show how femtosecond based x-ray probe methods can be used to detect the CIs in molecular systems. Therefore, we include only *S*_0_ and $\pi \sigma^{*}$ states, and the vibronic couplings of them with the other valence excited states have been neglected. These additional vibronic interactions^55,56,58,59^ and the other molecular modes like out-of-plane ring deformation,^54^ as mentioned above, may also play a role in describing the dissociation dynamics of pyrrole. Our model does not consider these effects. The treatment of all these couplings in an extended molecular mode space would be necessary for a fully quantitative description of the time-dependent photodissociation dynamics of pyrrole.^54–56,58,59^

**FIG. 1.**
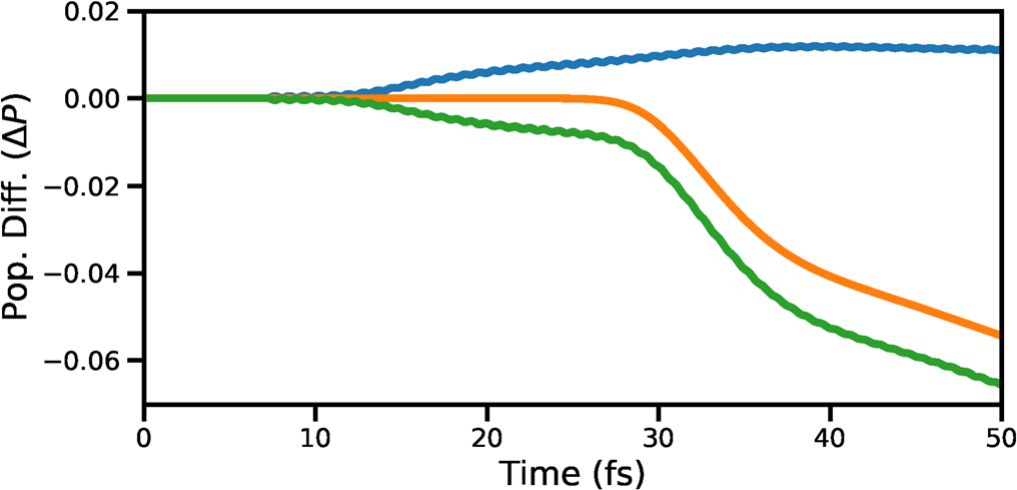
The plot shows the change in population ΔP after the pump-pulse ends for state *S*_0_ (blue curve), state $\pi \sigma^{*}$ (green curve), and the total population of the system (orange curve). When the wave-packets reach the vicinity of CI around 10 fs, the population branches out, and consequently, the population for the $\pi \sigma^{*}$ starts to decrease, and the population for *S*_0_ starts increasing. The system's total population starts to decay around 30 fs when the wave-packets hit the boundary of PES. The wave-packets are absorbed by the perfect-matched-layer placed at the edge of the PES.

**FIG. 2.**
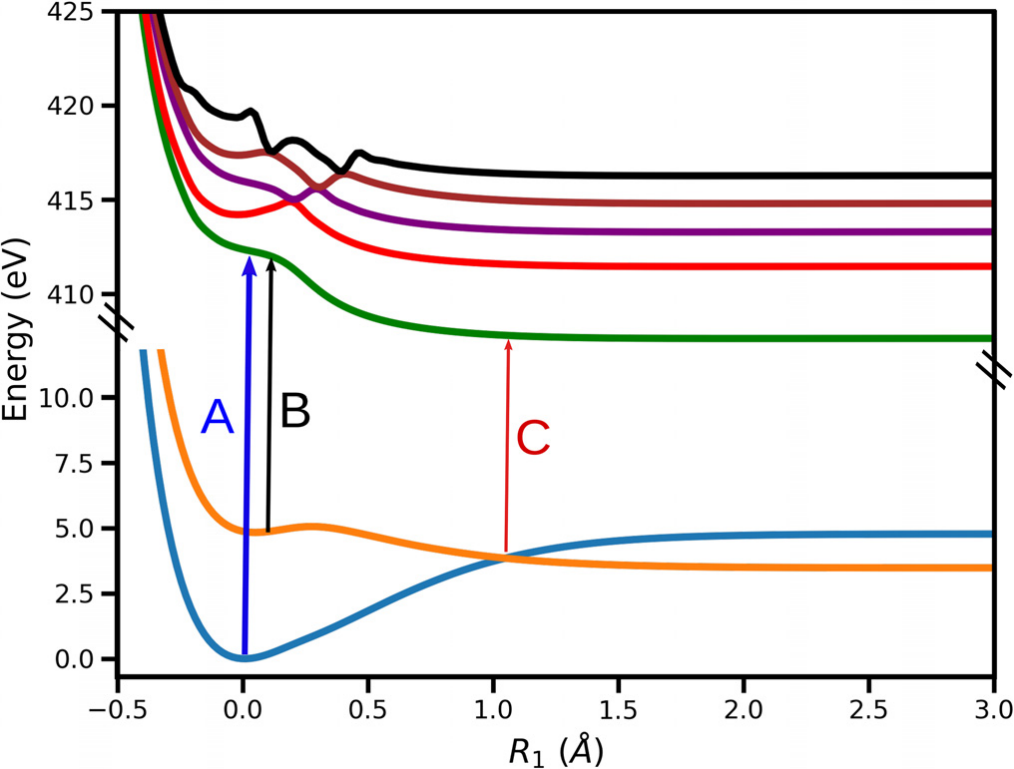
1D cuts of the PESs of *S*_0_ (blue), $\pi \sigma^{*}$ (orange) and five core-hole states (nitrogen 1s core-hole states) of the pyrrole molecule. These curves show variation of the energy of the states along the reaction coordinate *R*_1_. The transitions from the valence-states to the first core-hole state *C*_1_ are shown by various arrow. The transition between *S*_0_ and *C*_1_, represented by A, has an approximate energy of 412 eV. The transition represented by B between $\pi \sigma^{*}$ and *C*_1_ has an approximate energy of 407 eV (Ref. 60) and transition corresponding to the CI region is denoted by C and has an approximate energy of 404 eV.

A detailed discussion on the PESs of valence states employed in the current work can be found in a recent study investigating the cavity-modified dynamics of pyrrole.^57^ We provide the details very briefly here. Previous theoretical studies have established that the hydrogen elimination reaction dynamics in the $\pi \sigma^{*}$ state involves hydrogen in-plane and out-of-plane detachment motions.^29,57^ These coordinates, which will be denoted as *R*_1_ and *R*_2_, refer to the tuning and coupling coordinates, respectively. The computed PESs along the two reaction coordinates have been used to construct various spectroscopic signals from the quantum dynamics simulations. The valence states are considered to be in the diabatic basis. When the wave packets reach the CI, there is a diabatic population transfer (around 7% of population in the excited state), mediated by the diabatic coupling between the states. The wave-packets on the $\pi \sigma^{*}$ state PES arrive in the vicinity of CI at around 10 fs and then start to branch into *S*_0_, which can be seen in the population difference curves shown in Fig. 1 for the *S*_0_ state (blue curve) and $\pi \sigma^{*}$ state (green curve). The population difference for a particular state is calculated by subtracting the maximum population from the population at each time instance after the end of pump-pulse. The total population (orange curve) remains constant until the wave-packets reach the boundary of the PES and gets absorbed by the perfectly matched layer.^61^ To analyze the behavior of the system near CI, we use five core-hole states, which correspond to the nitrogen 1*s* orbital. The 1D strips of the PESs are shown in Fig. 2. The two valence states are denoted by *S*_0_ and $\pi \sigma^{*}$ while the lowest core-hole state is denoted by *C*_1_.

### B. Preparation of the system

**FIG. 3.**
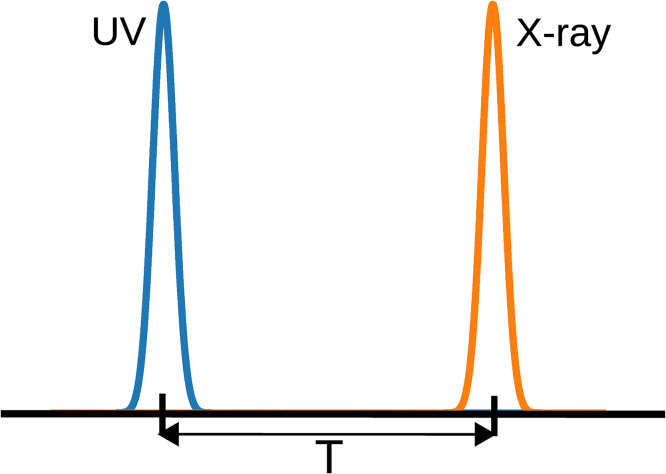
The schematic of the pump–probe scheme used for the construction of signals. Here, *T* is the delay time between the pump and the probe pulse.

A pump–probe scheme is used in the construction of the signals, as shown in Fig. 3. A UV pump-pulse is used to prepare the system by exciting it from the ground state (*S*_0_) to the valence excited state ($\pi \sigma^{*}$) of the molecule.

The interaction of the molecule with the pump-pulse is included in the initial Hamiltonian, and the system after the interaction is called the prepared system. The Hamiltonian of the corresponding system can be written as

$$\hat{H_{I}}={\hat{H}}_{0}+{\hat{H}}_{U},$$ (1)

where ${\hat{H}}_{0}$ is the molecular Hamiltonian for the valence states and ${\hat{H}}_{U}$ is the Hamiltonian, which considers the effects of pump-pulse on the molecule under the dipole approximation. The molecular Hamiltonian and the interaction with UV pump-pulse reads

$${\hat{H}}_{0}\;=\left[\begin{array}{cc} \hat{T}+{\hat{V}}_{0} & {\hat{C}}_{01}\\ {\hat{C}}_{01} & \hat{T}+{\hat{V}}_{1} \end{array}\right],$$ (2)

$${\hat{H}}_{U}\;=-E_{U}\cdot \;\text{cos}\;(\omega_{U}t)\cdot f_{U}(t)\left[\begin{array}{cc} 0 & {\hat{\mu }}_{01}\\ {\hat{\mu }}_{10} & 0 \end{array}\right].$$ (3)

In the Hamiltonian matrix in Eq. (2), $\hat{T}$ and $\hat{V}$ represent the kinetic energy operator and PES for a particular electronic state, respectively, and ${\hat{C}}_{01}$ represents the diabatic couplings between the states *S*_0_ and $\pi \sigma^{*}$. The reduced mass $m_{r}=1809.5\;a.u.$ is used in the kinetic energy operator $T=(-\hbar /2m_{r})\nabla^{2}$, with ∇ being the gradient operator with respect to the reaction coordinates *R*_1_ and *R*_2_. The *V*, *C*, and *μ* depend on the two reaction coordinates, *R*_1_ and *R*_2_. In the Hamiltonian describing the interaction with the pump, ${\hat{\mu }}_{01}$ and ${\hat{\mu }}_{10}$ represent the transition dipole moments between *S*_0_ and $\pi \sigma^{*}$ states. $E_{U}$ represents the electric field strength of pump-pulse, *ω_U_* is the carrier frequency of the pump-pulse, and $f_{U}(t)=\text{exp}\;(-t^{2}/2\sigma_{U}^{2})$ is the Gaussian envelope of the laser pulse.

## III. SPECTROSCOPIC SIGNALS

The expressions for the spectroscopic signals are discussed here, along with the modifications in Hamiltonian of the system. The electric field's effects from the pump pulse are calculated without any further approximation by including it in the system Hamiltonian (called prepared system) for all pump-probe signals.

### A. X-ray absorption signal

**FIG. 4.**
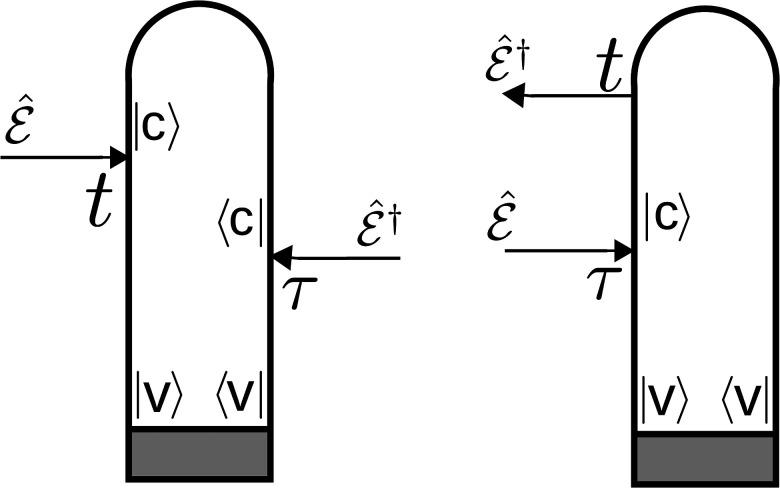
Loop diagrams corresponding to the XAS signal.^62,63^ The time runs along the loop from left bottom to right bottom. The interaction from left acts on the ket, and from the right acts on the bra. The arrows pointing inward represent the absorption of the photon. The gray region defines the system prepared by the pump pulse, |v⟩ represents the valence states, and |c⟩ represents the core-hole states.

In transient XAS, the absorption of an x-ray pulse, the interaction with a valence-to-core transition is recorded. The obtained spectrum contains information about the possible transitions. We note that photoelectrons can also be generated in a competing process. However, we neglect all the competing pathways and focus on the XAS signal. Here, we have included the pump pulse in the Hamiltonian, which creates the initial state [${\hat{H}}_{I}$ in Eq. (1)]. The interaction with the probe pulse is treated with time dependent perturbation theory. The total Hamiltonian can be written as follows:

$$\hat{H}(t)={\hat{H}}_{I}+{\hat{H}}_{\mathrm{int}}(t),$$ (4)

where ${\hat{H}}_{\mathrm{int}}(t)$ is the interaction Hamiltonian representing the interaction between probe-pulse and the prepared system. Under the rotating wave approximation (RWA), the time-dependent interaction Hamiltonian is

$${\hat{H}}_{\mathrm{int}}(t)={\hat{E}}_{X}(t){\hat{\mu }}_{X}^{†}+{\hat{E}}_{X}^{†}(t){\hat{\mu }}_{X},$$ (5)

with ${\hat{\mu }}_{X}\;({\hat{\mu }}_{X}^{†})$ being the transition dipole operator for describing the valence-to-core transition and ${\hat{E}}_{X}(t)$ is the electric field operator of the x-ray modes. The field state is considered to be a coherent state initially. The signal for the XAS spectrum is defined as the integrated rate of change of the number of photons as a function of the absorption frequency *ω_s_* and a pump–probe delay *T* and can be expressed as follows:

$$S(T,\omega_{s})=\int_{-\infty }^{\infty }dt\frac{d\langle N_{s}\rangle }{dt},$$ (6)

with *N_s_* being the photon number operator for the *s*th field-mode. The interaction Hamiltonian, ${\hat{H}}_{\mathrm{int}}(t)$ in Eq. (4), appears in the signal expression as a consequence of the probe-pulse electric field ${\hat{E}}_{X}$. The signal expression is then obtained by treating ${\hat{H}}_{\mathrm{int}}(t)$ as the time-dependent perturbation. The expression then reads^27,64–66^

$$\begin{array}{c} S(T,\omega_{s})=\frac{2}{\hbar^{2}}E^{*}(\omega_{s})[\int_{-\infty }^{\infty }e^{i\omega_{s}(t-T)}dt\\ \times \int_{-\infty }^{\infty }d\tau E_{0}(\tau -T)e^{-i\omega_{X}(\tau -T)}C_{S}(t,\tau )] \end{array},$$ (7)

where *ω_X_* is the carrier frequency of the x-ray probe-pulse, $E_{0}(t)=\text{exp}\;(-t^{2}/2\sigma_{X}^{2})$ is its Gaussian envelope, and *ω_s_* is the frequency of the *s*th mode of the probe, and *C_S_* is the correlation function, which contains the information about the interaction between the probe-pulse and the system, for time instances *τ* and *t* as shown in Fig. 4,

$$C_{S}(t,\tau )=\langle \psi_{0}|\hat{\mu }(t){\hat{\mu }}^{†}(\tau )|\psi_{0}\rangle ,$$ (8)

where $\left|\psi_{0}\right\rangle$ represents the superposition of the wave functions that evolve in the valence states of the molecule according to the prepared-system Hamiltonian ${\hat{H}}_{I}$. Note that Eq. (7) describes both absorption and stimulated emission according to the diagrams in Fig. 4.

### B. X-ray Spontaneous Emission Signal

The probe pulse of a particular center frequency is used to excite the system from the valence states to the core-hole states. The 1s core-hole states of nitrogen in pyrrole have a lifetime of ≈ 7 fs (Refs. 32–34), and hence spontaneous emission back to the valence states occurs on this timescale. To keep the computational effort tractable and stay within Hilbert space, we introduce the core-hole lifetime as an empirical parameter. Here, we also neglect the photoionization due to the x-ray probe pulse as well as the subsequent Auger decay^67–69^ and instead solely focus on the spontaneous emission process. Note that the Auger process can be a dominant decay channel for relaxation from core excited states in light elements.^70,71^ The total Hamiltonian for constructing the XSES signal is similar to the Hamiltonian in Eq. (4). The difference is that now both the pump and probe pulses are included in the initial Hamiltonian, and the subsequent spontaneous emission process is treated with perturbation theory. The molecular Hamiltonian, the pump interaction, and probe interaction are expressed as follows:

$${\hat{H}}_{0}=\left[\begin{array}{ccc} \hat{T}+{\hat{V}}_{0} & {\hat{C}}_{01} & 0\\ {\hat{C}}_{01} & \hat{T}+{\hat{V}}_{1} & 0\\ 0 & 0 & \hat{T}+{\hat{V}}_{2} \end{array}\right],$$ (9)

$${\hat{H}}_{U}=-E_{U}\cdot \;\text{cos}\;(\omega_{U}t)\cdot f_{U}(t)\left[\begin{array}{ccc} 0 & {\hat{\mu }}_{01} & 0\\ {\hat{\mu }}_{10} & 0 & 0\\ 0 & 0 & 0 \end{array}\right],$$ (10)

$${\hat{H}}_{X}=-E_{X}\cdot \;\text{cos}\;(\omega_{X}t)\cdot f_{X}(t)\left[\begin{array}{ccc} 0 & 0 & {\hat{\mu }}_{02}\\ 0 & 0 & {\hat{\mu }}_{12}\\ {\hat{\mu }}_{20} & {\hat{\mu }}_{21} & 0 \end{array}\right].$$ (11)

**FIG. 5.**
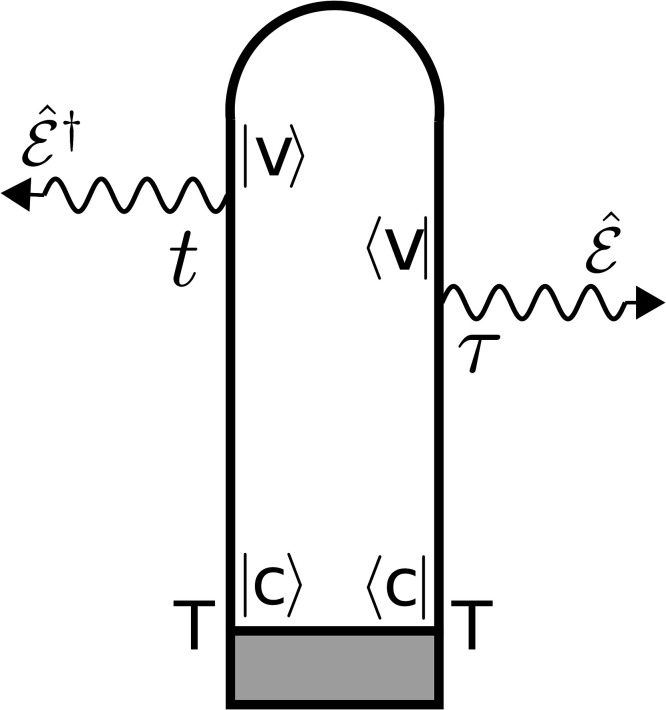
Loop diagram corresponding to the XSES. The gray region defines the interaction of system with pump-pulse as well as probe-pulse, and T is the time-delay between the pump-pulse and the probe-pulse.

The notations in the molecular and pump field Hamiltonians are identical to the notation used in Eqs. (2) and (3) with ${\hat{V}}_{2}$ being a core-hole state. In the probe field, Hamiltonian matrix ${\hat{H}}_{X},\;\hat{\mu }$'s represents the dipole moment between the valence (*S*_0_ and $\pi \sigma^{*}$) and core-hole (*C*_1_) states, $E_{X}=$ 2.06 $\times 10^{11}$ V/m is the electric field strength of the probe pulse, *ω_X_* is the carrier frequency of probe-pulse in x-ray regime, and $f_{X}(t)=\text{exp}\;(-t^{2}/2\sigma_{X}^{2})$ is the Gaussian envelope of the probe-pulse. The field operator of the spontaneously emitted photons takes the following form:

$$\hat{E}(t)=\sum_{s}{\left(\frac{2\pi \hbar \omega_{s}}{\Omega }\right)}^{1/2}{\hat{a}}_{s}e^{-i\omega_{s}t},$$ (12)

where Ω is the quantization volume, and *ω_s_* represents the frequency of the spontaneously emitted photon. The XSES signal is defined as the integrated rate of change of the number of photons as a function of the emission frequency *ω_s_* and the pump-probe delay *T*. The signal expression then reads^72^

$$\begin{array}{c} S(T,\omega_{s})=-\frac{2}{\hbar \pi c^{3}}ℜ[\int_{-\infty }^{\infty }dt\int_{0}^{\infty }\omega_{s}^{3}d\omega_{s}\int_{-\infty }^{t}d\tau \times e^{i\omega_{s}(t-\tau )}e^{-\gamma t}C_{E}(\tau ,t)], \end{array}$$ (13)

where γ=1/δt, with δt= 7 fs, is the decay rate for the core-hole states and *C_E_* is the correlation function corresponding to the diagram shown in Fig. 5. The integral over the frequency *ω_s_* is skipped in the final signal calculations as it is the variable for the frequency resolved detection. The two-time correlation function can be written as follows:

$$C_{E}(\tau ,t)=\langle \psi_{0}(T)|{\hat{\mu }}^{†}(\tau )\hat{\mu }(t)|\psi_{0}(T)\rangle ,$$ (14)

where $\left|\psi_{0}(T)\right\rangle$ represents the *C*_1_ state, which is populated at the pump–probe delay *T* by the x-ray pulse.

### C. TRUECARS

**FIG. 6.**
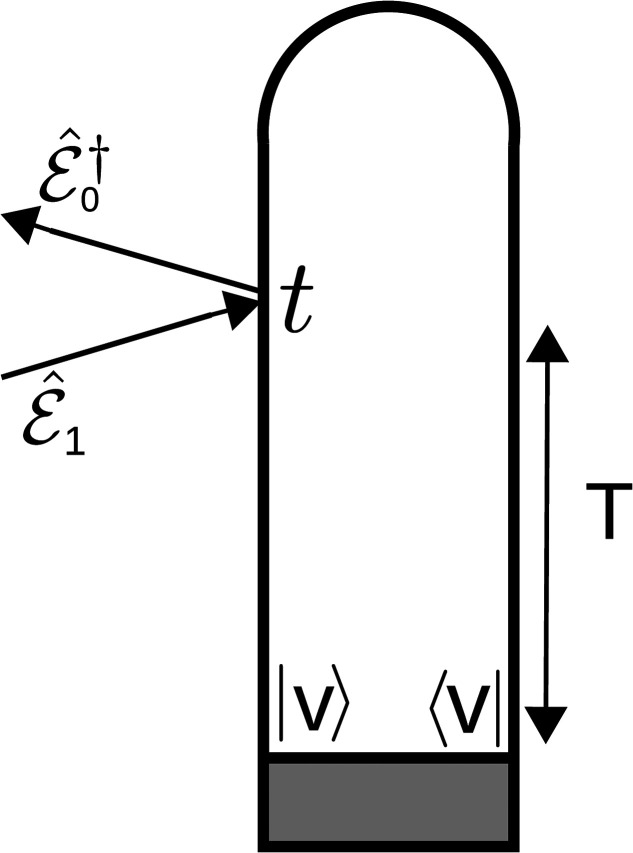
Loop diagram corresponding to the TRUECARS signal. The gray region defines the system prepared by the pump pulse, |v⟩ represents the valence states, and T defines the time-delay between the pump-pulse and the hybrid probe-pulses.

In contrast to XAS and XSES, TRUECARS is a technique that is only sensitive to the vibronic coherences generated in the molecule. It uses a hybrid, off-resonant probe-pulse sequence. The signal consists of a Stokes and anti-Stokes type signal generated due to the redistribution of the photons between different modes. The TRUECARS spectrum is centered at the difference of the carrier frequencies of the hybrid probe pulses. The Raman shift indicates the energy difference between the involved valence states. Here, we assume that the probe pulses have the same carrier frequency. The loop diagram corresponding to the TRUECARS signal is shown in Fig. 6. The TRUECARS signal, which is constructed by perturbative treatment of the hybrid probe pulses, is represented by the following expression:^38^

$$\begin{array}{c} S(\omega_{r},T)=2ℑ\int_{-\infty }^{\infty }dte^{i\omega_{r}(t-T)}E_{0}^{*}(\omega_{r})\\ \times E_{1}(t-T)\langle \psi (t)|\hat{\alpha }|\psi (t)\rangle \end{array},$$ (15)

where ψ(t) is the linear combination of the valence-states wave function at the pump–probe delay *T*, *ω_r_* is the Raman frequency, $E_{0}$ and $E_{1}$ are the two probe pulses, respectively, and $\hat{\alpha }$ represents the polarizability tensor of the molecule:^73^

$$\hat{\alpha }=\left[\begin{array}{ccc} \alpha_{xx} & \alpha_{xy} & \alpha_{xz}\\ \alpha_{yx} & \alpha_{yy} & \alpha_{yz}\\ \alpha_{zx} & \alpha_{zy} & \alpha_{zz} \end{array}\right],$$ (16)

where each element *α_ij_*, with i,j=x,y,z represents the directions of polarization. Each tensor element is expanded on the basis of the valence states:

$${\hat{\alpha }}_{ij}=\left[\begin{array}{cc} \alpha^{00} & \alpha^{01}\\ \alpha^{10} & \alpha^{11} \end{array}\right].$$ (17)

Every element *α^kn^*, with *k*, *n* representing the initial and final valence states, depends on the nuclear coordinates *R*_1_ and *R*_2_, and the frequency of the probe-pulses *ω:*^73^

$$\alpha_{ij}^{kn}=\frac{1}{\hbar }\sum_{r}(\frac{\mu_{i,kr}\mu_{j,rn}}{\omega_{rn}-\omega }+\frac{\mu_{i,rn}\mu_{j,kr}}{\omega_{rk}+\omega }),$$ (18)

where $\omega_{rn}=(E_{r}-E_{n})/\hbar$ is the resonance frequency between the core-hole state *r* and the valence state *n*.

The matrix from Eq. (17) becomes non-Hermitian when Eq. (18) is used. Here, we make the assumption that the energy difference between the valence states is small compared to $\omega_{rn}-\omega$ and the off diagonal elements can be written as the average: $\alpha^{\prime }=(\alpha^{01}+\alpha^{10})/2$. The new polarizability matrix, that is used in the calculations, then reads

$${\hat{\alpha }}_{ij}^{\prime }=\left[\begin{array}{cc} \alpha^{00} & \alpha^{\prime }\\ \alpha^{\prime } & \alpha^{11} \end{array}\right].$$ (19)

## IV. METHODS

The ground state minimum structure of pyrrole, optimized at the DFT/B3LYP/aug-cc-pVDZ level of theory, has been used as the reference geometry for computing the PESs. A rigid 2D-PES scan has been performed by employing the complete self-consistent field (CASSCF) method along with the aug-cc-pVDZ basis set. Three states have been included in the state-averaged CASSCF for calculating the PESs corresponding to the electronic valence states. The active space is composed of seven orbitals (five *π*-orbitals of ring and a pair of $\sigma /\sigma^{*}$-orbitals of NH bonds) and eight electrons. To compute the core excited state energies involving the electronic transition from the 1s-orbital of nitrogen atom, the complete active space configuration interaction (CASCI) method has been used. The prior-mentioned 3-state averaged CASSCF wave function and the corresponding orbitals have been utilized to generate the configurations in the CASCI method. The diabatic PESs have been obtained using the quasi-diabatization procedure as implemented in MOLPRO-2019. The corresponding transformation matrix has been used to transform the dipole moments from adiabatic to diabatic basis. The program package MOLPRO-2019^74,75^ has been used for the electronic structure calculations.

The correlation functions in Eqs. (8), (14), and (15) have been calculated by a direct propagation simulation protocol.^76^ The wave packet dynamics have been calculated with our in-house software QDng. The Arnoldi method^77^ was used to propagate the wave functions. The PESs and the numerical wave functions are represented on a grid with dimensions 256 × 256. A perfectly matched layer is used to absorb the wave packets at the boundary of the numerical grid. The second derivative with respect to the reaction coordinates is calculated using the Fourier transform method. For all the propagations, a time step size of 50 has been used. Since the lifetime of nitrogen 1s core-hole states is ≈ 7 fs, the time-evolution of the core-hole states in the XSES signal is done until 12.2 fs.

## V. RESULTS AND DISCUSSION

**FIG. 7.**
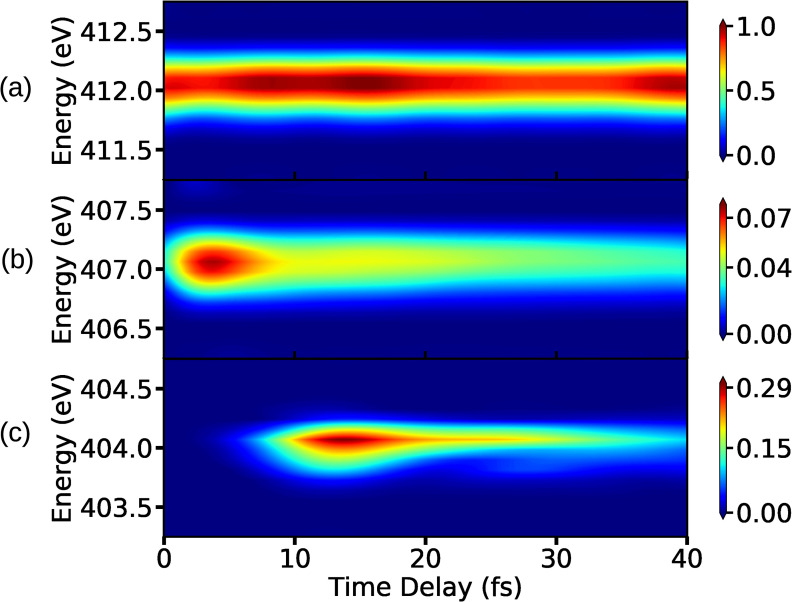
The XAS spectra for the lowest core-hole for x-ray center frequencies with (a) $\omega_{X}=$ 412 eV, (b) $\omega_{X}=$ 407 eV, and (c) $\omega_{X}=$ 404 eV. The other UV pulse and x-ray pulse parameters used for the signal calculations are as follows: $E_{U}=$ 7.71 $\times 10^{10}$ V/m, $\sigma_{U}=$ 1 fs (FWHM= 2.35 fs), $\omega_{U}=$ 4.76 eV, and $\sigma_{X}=$ 1.5 fs (FWHM= 3.53 fs).

The XAS signal is calculated by solving Eq. (7) for the core-hole states shown in Fig. 2. The *C*_1_ state is energetically 3−4 eV apart from nearest core-hole state in the vicinity of the CI and thus the XAS signal is constructed only for the *C*_1_ state. This can be used to distinguish the various features in the spectrum. The x-ray pulse has a finite width in the energy domain, and thus the signal can be expected to show sharp features. Using an appropriate pulse width and center-frequency of the x-ray pulse, it is possible to probe absorption bands belonging to different energy regimes as shown in Fig. 2. The signals are shown in Fig. 7 for three different x-ray probe center frequencies, corresponding to transitions A, B, and C in Fig. 2. Note that, when all the core-hole states are considered for the signal construction, the transitions corresponding to A and B for the *C*_1_ state will be resonant with transitions from other core-hole states as well. The XAS spectrum shown in Fig. 7(a) corresponds to the transition from state *S*_0_ to state *C*_1_ in the energy regime denoted by A in Fig. 2. The signal has almost constant intensity for all measured time-delays, while small variations in intensity originate from the pump pulse and the unbalanced branching ratio near the CI. We note that the signal shows similar features as the attosecond transient absorption spectroscopy spectrum recorded for the carbon K-edge in $C_{2}H_{4}^{+}$ to study the conical intersection between the *D*_0_ and *D*_1_ states.^25^ This signal has higher intensity compared to signals corresponding to the transitions B and C in Fig. 2. The reason being the population residing at the local minima of *S*_0_ state, as shown in Fig. 2, is higher than the population transferred to the $\pi \sigma^{*}$ state by the pump pulse. Hence, the intensity of signal in Fig. 7(a) is used as a reference for signals corresponding to other energy regions. The signal shown in Fig. 7(b) corresponds to the transition B in Fig. 2, and it has highest intensity when pump-pulse is about to vanish and the intensity decreases when the wave packets starts to move away from the FC point. The steady decrease in the intensity after some time indicates the presence of a local minimum in the $\pi \sigma^{*}$ state. A certain amount of population is trapped in that minima that continues to leak over time. Figure 7(c) indicates that the absorption is taking place near the CI: the signals appears when the wave-packets on the $\pi \sigma^{*}$ state arrive in the vicinity of the CI and it vanishes when the wave-packets move past the CI region and move out of resonance with the probe-pulse. The transition from the $\pi \sigma^{*}$ state to *C*_1_ near the CI has a significantly higher intensity compared to transition between the same states in the FC region (transition B in Fig. 2), and it can be observed by comparing Figs. 7(b) and 7(c). The increase in the intensity of signal near the CI indicates that the transition dipole-moment between the involved states increases in the vicinity of the CI. The fact that there are parts of the wave packets retained in the local minimum at the FC of the $\pi \sigma^{*}$ state supports the conclusion about increase in transition dipole-moment around CI. There is an increase in the intensity of the signal in Fig. 7(a), between 5 and 20 fs and around 40 fs, when the wave packets pass through the CI. It is a result of the transfer of low energy wave-packets from $\pi \sigma^{*}$ to the *S*_0_ state near CI that return to the minima of *S*_0_ state. These vibrationally excited wave packets oscillate in the bounded region of the *S*_0_ potential. Note that it is not possible to observe the change in the nature of the $\sigma^{*}$ orbital along the dissociation coordinate in XAS spectrum. The x-ray probe frequency required to probe the transformation region is almost resonant with the absorption frequency of the CI region. As a result, the XAS signal from the CI region dominates the XAS signal from the Rydberg-to-valence transformation region.

**FIG. 8.**
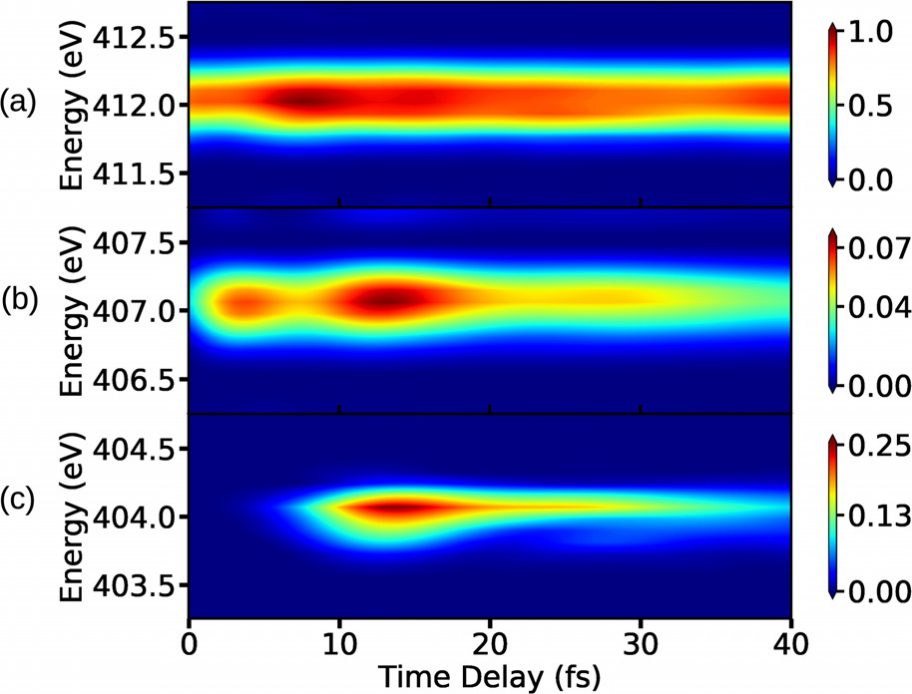
(a) The XAS spectra for five core-hole states for (a) $\omega_{X}=$ 412 eV, (b) $\omega_{X}=$ 407 eV, and (c) $\omega_{X}=$ 404 eV. The other pulse parameters for the UV pulse and x-ray pulse are the same as the ones used in Fig. 7. The bump in (b) appears because the CI region is resonant with *C*_2_ for $\omega_{X}=$ 407 eV, which was not present in Fig. 7(b).

The XAS spectra shown in Fig. 8 contain the contributions from five core-hole states. The non-adiabatic couplings between the core-hole states, which have avoided crossings, are not included in the calculations. The avoided crossings are spatially well separated from the CI between the valence states, and thus it is expected that these crossings do not significantly alter the spectrum. When all five core-hole states (shown in Fig. 2) are used to construct the XAS signal, multiple valence-to-core transitions can be resonant with the same probe-pulse center-frequency. The transition C in Fig. 2 has the lowest energy, and it is the only transition resonant with the probe pulse with a center frequency 404 eV. The number of resonant transitions increases by further increasing the carrier frequency.

The XAS signal corresponding to the transitions taking place in the vicinity of CI is shown in Fig. 8(c). Due to the resonance condition, the signal in Fig. 8(c) has the same shape as the signal for a single core-hole state in Fig. 7(c). The transition corresponding to the FC region for the $\pi \sigma^{*}$ state is resonant with the transition between the *C*_2_ state and the CI region, and hence there is an increase in the intensity of the signal between 10 fs and 20 fs in Fig. 8(b). The overlap of multiple signals can also be seen in Fig. 8(a), which correspond to the absorption from the *S*_0_ state's local minimum. Since the intensity of the signal in Fig. 8(a) is used as a reference for other [Figs. 8(b) and 8(c)] signals, the spectrum in Fig. 8(c) has a lower intensity than the signal in Fig. 7(c). Apart from the change in peak intensity for absorption corresponding to 412 eV in Figs. 7 and 8, there is a noticeable difference between the shapes of the signals as well. The increase in intensity in the XAS spectrum in Fig. 8(c) at around 8 fs can be interpreted as an arrival of the wave packet in the CI region.

The XSES spectrum can be used to complement the findings from the XAS signal and to observe the energy gap between the valence states directly. The XSES spectrum is constructed by solving Eq. (13). For the sake of clarity, the XSES signals are constructed for both *S*_0_ and $\pi \sigma^{*}$ states separately. In the simulated spectra, the contribution to the total signal from each state can be evaluated.

**FIG. 9.**
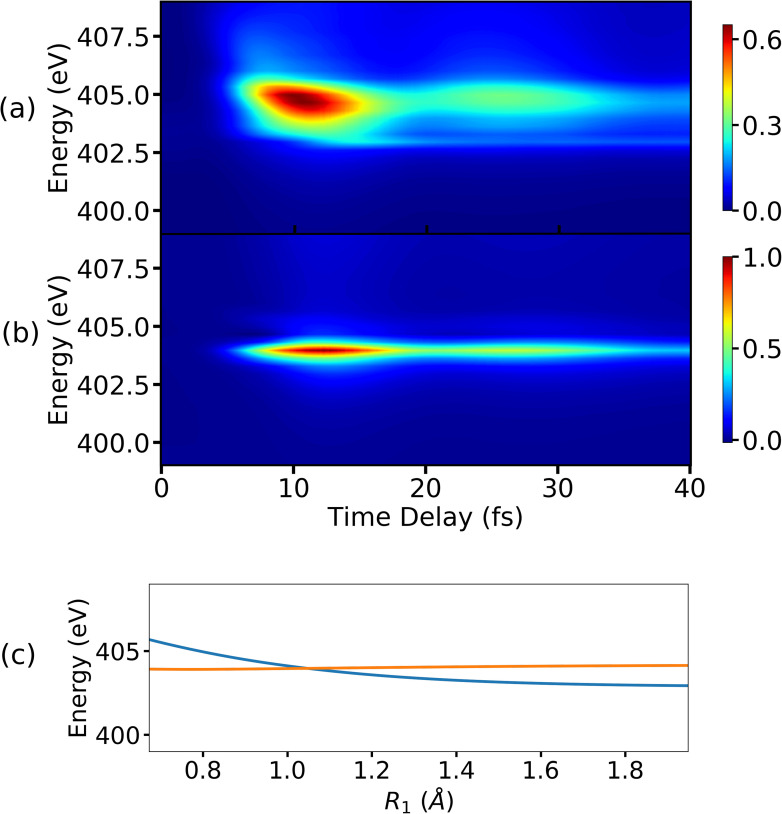
(a) The *S*_0_ state contribution to the XSES spectrum for the *C*_1_ state. (b) The $\pi \sigma^{*}$ state contribution to the XSES spectrum. (c) The separation between the *S*_0_ and *C*_1_ state (blue curve), and separation between $\pi \sigma^{*}$ and *C*_1_ state (orange curve) as a function of the reaction coordinate *R*_1_ for a fixed value of other reaction coordinate *R*_2_ is shown here. The UV pulse and x-ray pulse parameters used for the signal calculations are as follows: $E_{U}=$ 7.71 $\times 10^{10}$ V/m, $\sigma_{U}=$ 1 fs (FWHM= 2.35 fs), $\omega_{U}=$ 4.76 eV, and $E_{X}=$ 2.06 $\times 10^{11}$ V/m, $\sigma_{X}=$ 0.5 fs (FWHM= 1.18 fs), and $\omega_{X}=$ 403.3 eV.

The spectrum shown in Fig. 9(a) corresponds to the signal of the *S*_0_ final state and it gets stronger when the wave packets approach the CI. The signal decays when the location of the wave packets is not resonant anymore with the probe pulse. This behavior follows a trend between 5 fs and 20 fs, which corresponds to the blue curve in the Fig. 9(c) representing the separation between the *S*_0_ state and the *C*_1_ state as a function of a reaction coordinate. The signal that corresponds to the $\pi \sigma^{*}$ state is shown in Fig. 9(b), and the increase and decrease in intensity over the time show a similar behavior as in the case of the *S*_0_ state.

The change in intensity as a function of emission frequency and time delay in XSES spectra can be understood as follows: The spatial position of the wave packets on a PES relates them to a possible transition frequency for a particular state. If the energy of an excitation pulse is resonant or close to resonance for that transition frequency, the excitations will be maximum which would result in higher emission. Since the energy of the x-ray pulse is 403.3 eV, the signal will have the highest intensity when the wave packets reach the region before the CI which has resonance frequency of around 404 eV. For a specific delay, the strength of the signal would depend upon the transition dipole moment between the valence and core-hole states. Out of the two valence states, the $\pi \sigma^{*}$ state has higher amplitude of transition dipole-moment to the *C*_1_ state and hence will have higher intensity of XSES signal.

**FIG. 10.**
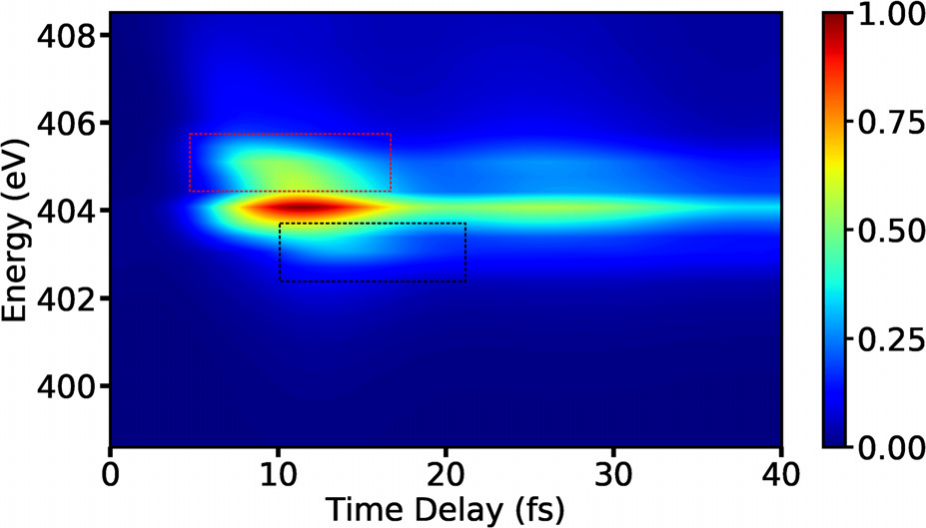
The total XSES spectrum for the *C*_1_ state. The regions marked with red and black dotted boxes correspond to the *S*_0_ state PES before and after the CI, respectively.

As can be seen in Fig. 9(c), the separation between the $\pi \sigma^{*}$ state and the *C*_1_ state is almost constant (represented by an orange curve) and a similar feature is found in the XSES spectrum in Fig. 9(b). This indicates that the XSES may be able to project the relative shapes of the PESs of involved states. The total XSES spectrum, which includes both the valence states, is shown in Fig. 10. The region shown in the red box corresponds to the *S*_0_ state before the CI, and the region shown in the black box corresponds to the same state but after the CI. The signal in the black box starts to develop around 10 fs, which matches the time when the system reaches the CI as predicted by the XAS signal in Fig. 7. The valence states can be seen to approach each other before 10 fs and move apart subsequently.

Assuming that the transition dipole moments between the two valence states and the core-hole states are comparable to each other, the XSES signal can be used to detect the two PESs approaching each other before the CI and move apart after the CI. The only necessary condition is that the energy of the x-ray pulse should be resonant with the core-hole states in CI region.

**FIG. 11.**
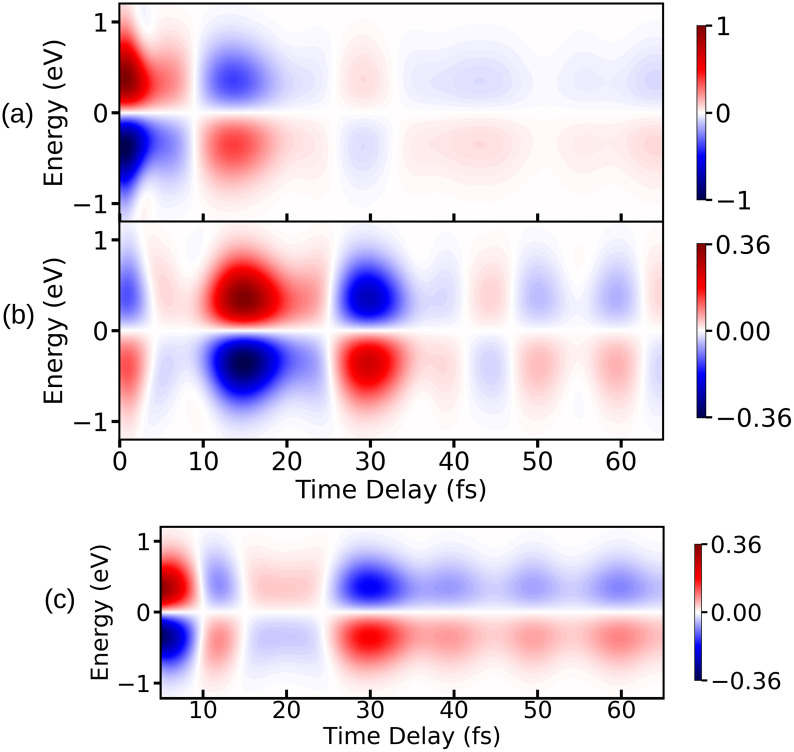
The TRUECARS spectra for (a) *α_xx_* and (b) *α_yy_* (c) *α_xx_* + *α_yy_* are shown here. Note that the time-delay axis in (c) is cropped before 5 fs to enhance the features of the signal. The intensity axes for all the figures are normalized with respect to the signal with highest intensity for the sake of comparison. The UV pulse parameters are as follows: $E_{U}=$ 2.06 $\times 10^{10}$ V/m, $\sigma_{U}=$ 1.2 fs, and $\omega_{U}=$ 4.76 eV. The parameters for the first probe-pulse $E_{0}$ and second probe-pulse $E_{1}$ are as follows: $\sigma_{0}=$ 0.9 fs (FWHM= 2.12 fs), $\omega_{X}=$ 401 eV, and $\sigma_{1}=$ 1.7 fs (FWHM= 4 fs).

We now discuss the TRUECARS spectrum. Figure 11 shows the TRUECARS spectrum, which has been calculated using Eqs. (15) and (19). The TRUECARS spectra for the *xx* and *yy* components of the x-ray polarizability tensor, *α_xx_* and *α_yy_*, are shown separately in Figs. 11(a) and 11(b) along with the sum of both components in Fig. 11(c). Note that the sum spectrum corresponds to a rotational averaged spectrum, which would be observed in a gas-phase experiment. The spectrum corresponding to *α_zz_* is not shown here because it is five orders of magnitude weaker. The TRUECARS spectrum without the UV pump pulse has been subtracted from that of the prepared system with the UV pump pulse to remove the contribution from ground state vibrational Raman transitions.

The peak before 5 fs stems from the vibrational coherences created by the pump-pulse. At around 5–10 fs, the wave packet reaches the CI region where the non-adiabatic couplings come into effect. Inspection of Figs. 11(a) and 11(b) shows an increase in intensity, indicating a build up of coherence.

**FIG. 12.**
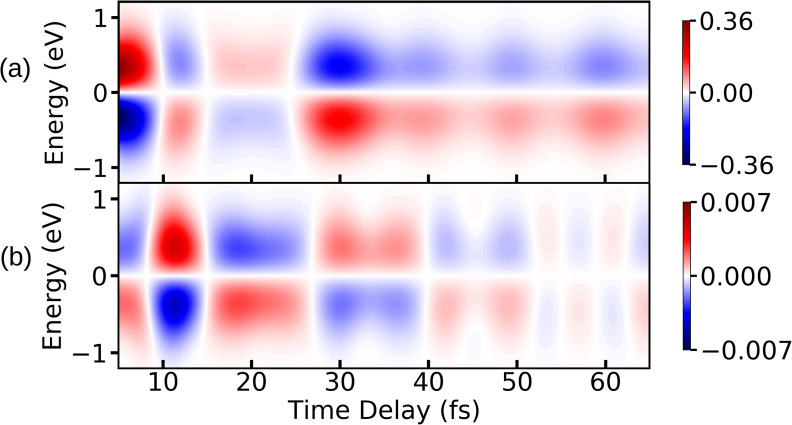
The TRUECARS signal for (a) vibrational coherence and (b) electronic coherence are shown. The intensity axes are normalized with respect to the TRUECARS signal in Fig. 11(a).

Between 10 and 30 fs, the effects of the CI can be seen in the spectrum. Both components in Figs. 11(a) and 11(b) show an increase in intensity. The *xx* component in Fig. 11(a) peaks between 10 and 20 fs and falls off at later times. The *yy* component in Fig. 11(b) also peaks between 10 and 20 fs but has another strong peak at ≈ 30 fs. This increase can be explained by the buildup in coherence as well as an increase in polarizability. The *xx* component of the polarizability of the $\pi \sigma^{*}$ peaks before the CI (at short NH bond distances) and goes to nearly zero after the wave packet has passed through the CI. The *yy* component shows the opposite behavior: it is near zero before the wave packet reaches the CI and peaks at larger NH bond distances. This explains the peak in Fig. 11(b) at 30 fs. The Stokes and anti-Stokes patterns of the spectra for the *xx* and *yy* components are out of phase, leading to a partial cancelation of the signal in Fig. 11(c). However, the oscillation patterns still have the same period. The strength of the TRUECARS signal depends on the magnitude of the matrix elements of the polarizability operator [see Eq. (19)] along with the magnitude of the coherence generated. The diagonal elements of the polarizability operator constitute the vibrational coherence, while the off diagonal elements constitute the electronic coherence in the spectrum. The TRUECARS spectra are constructed separately for the vibrational coherence and the electronic coherence to compare the effect on the overall signal. The TRUECARS signal corresponding to electronic coherence is shown in Fig. 12(b), and it has a maximum intensity around 10 fs, which is the time-instance when the wave packets start reaching the CI. The TRUECARS signal corresponding to the vibrational coherence is shown in Fig. 12(a). Inspection of the polariziabilty matrix elements [see Eq. (19)] also reveals that the diagonal elements are ≈ 2 times larger than the off diagonal elements. Thus, the electronic coherences contribute significantly less than the vibrational coherences to the overall TRUECARS signal. Hence, the key signature of the CI, i.e., electronic coherence generation, is not clearly visible in the TRUECARS signal for pyrrole. Note that the vibrational and electronic degrees of freedom are strongly mixed in the direct vicinity of the CI and thus one cannot clearly distinguish between vibrational and electronic coherences.

After 30 fs, the intensity of the signal decreases due to the dissociation. Note that this decay is mainly caused by absorption of the wave packet at the grid boundary (see Fig. 1). In these particular examples, the TRUECARS spectrum does not show the expected frequency resolution and time evolution of the energy gap between the valence states cannot be visualized. This is mainly due to the choice of pulse parameters, which are necessary to resolve the spectral features in the time domain.

## VI. CONCLUSION

We have studied the CI in pyrrole using three different spectroscopy techniques, namely XAS, XSES, and TRUECARS. A set of transient XAS spectra was constructed by selecting the dominant states involved in the non-adiabatic dynamics (*S*_0_, $\pi \sigma^{*}$) and the transitions to the first nitrogen 1s core hole state. We could show that the signature of the CI in the spectrum consists of a resonance and an increase in signal intensity: For the lowest probe energy, the probe becomes resonant with the first core-hole state, and a peak appears in the spectrum when the wave packet reaches the CI region. The transition dipole moment peaks in the vicinity of the CI leading to an increased absorption indicating the time it takes for the wave packet to reach the CI, allowing us to narrow down the timing further. Due to the unbalanced branching of the wave packet at the CI, we see mainly the signature of the $\pi \sigma^{*}$ state in spectrum.

The time-resolved XSES spectrum was constructed using an x-ray probe-pulse, which has a frequency that is resonant with the valence-to-core transition corresponding to the CI region. The $\pi \sigma^{*}$ state and the *C*_1_ state have a similar shape and thus a constant energy difference in the vicinity of CI. Consequently, the spontaneous emission peaks at a rather constant photon energy. In contrast to the XAS spectrum, the branching ratio at the CI is not a decisive factor. The spontaneous decay from the nitrogen 1s core hole state has a similar transition moment to *S*_0_ and the $\pi \sigma^{*}$ state. As a result, one can now see a signature of the curve crossing in the XSES spectrum.

For the third technique, we have constructed the TRUECARS signal. Here, we explicitly probe the vibronic coherences that are generated in the molecule by means of a linear off-resonant Raman process. The TRUECARS signal depends on the x-ray transition polarizability and the magnitude of the electronic and vibrational coherences of the excited system. The simulated spectrum displays peaks between 10 fs and 20 fs, which hint at non-adiabatic dynamics due to the CI. The spectra originating from the two polarizability tensor components are out of phase, and the overall signal is diminished. The spectral features correlate with the signatures of the XAS and XSES spectra putting the passage through the CI consistently between 10 fs and 20 fs. We note that the TRUECARS spectrum is dominated by the vibrational coherences and the increase in the polarizability near the CI rather than the electronic coherences.

In conclusion, we have shown that the existence of a CI in a molecule and its temporal appearance can be identified by a combination of multiple methods. Each technique on its own may deliver a spectrum that could potentially be difficult to interpret. The presented techniques probe different properties of the molecule and thus their combination gives a more complete picture. We also note that the combination is also a potential strategy to bypass the high demand on the pulse bandwidth to map out the rapidly changing energy near the CI. The pulses used in our simulations were longer than 1 fs, thus explicitly avoiding attosecond pulses.

## Data Availability

The data that support the findings of this study are available from the corresponding author upon reasonable request.
